# Revisiting gliomatosis cerebri in adult-type diffuse gliomas: a comprehensive imaging, genomic and clinical analysis

**DOI:** 10.1186/s40478-024-01832-w

**Published:** 2024-08-10

**Authors:** Ilah Shin, Yae Won Park, Yongsik Sim, Seo Hee Choi, Sung Soo Ahn, Jong Hee Chang, Se Hoon Kim, Seung-Koo Lee, Rajan Jain

**Affiliations:** 1 Department of Radiology, Seoul St. Mary’s Hospital, College of Medicine, The Catholic University of Korea, 222 Banpo-daero, Seocho-gu, Seoul, 06591 Republic of Korea; 2 Department of Radiology and Research Institute of Radiological Science and Center for Clinical Imaging Data Science, College of Medicine, Yonsei University, 50 Yonsei-ro, Sedaemun-gu, Seoul, 03722 Republic of Korea; 3 Department of Radiation Oncology, Yonsei University College of Medicine, 50 Yonsei-ro, Sedaemun-gu, Seoul, 03722 Republic of Korea; 4 Department of Neurosurgery, Yonsei University College of Medicine, 50 Yonsei-ro, Sedaemun- gu, Seoul, 03722 Republic of Korea; 5 Department of Pathology, Yonsei University College of Medicine, 50 Yonsei-ro, Sedaemun-gu, Seoul, 03722 Republic of Korea; 6 Department of Radiology, New York University Grossman School of Medicine, 550 1st Ave, New York, NY States USA; 7 Department of Neurosurgery, New York University Grossman School of Medicine, 550 1st Ave, New York, NY States USA

**Keywords:** Glioma, Glioblastoma, Gliomatosis cerebri, Magnetic resonance imaging, World Health Organization

## Abstract

Although gliomatosis cerebri (GC) has been removed as an independent tumor type from the WHO classification, its extensive infiltrative pattern may harbor a unique biological behavior. However, the clinical implication of GC in the context of the 2021 WHO classification is yet to be unveiled. This study investigated the incidence, clinicopathologic and imaging correlations, and prognostic implications of GC in adult-type diffuse glioma patients. Retrospective chart and imaging review of 1,211 adult-type diffuse glioma patients from a single institution between 2005 and 2021 was performed. Among 1,211 adult-type diffuse glioma patients, there were 99 **(**8.2%) patients with GC. The proportion of molecular types significantly differed between patients with and without GC (*P* = 0.017); IDH-wildtype glioblastoma was more common (77.8% vs. 66.5%), while IDH-mutant astrocytoma (16.2% vs. 16.9%) and oligodendroglioma (6.1% vs. 16.5%) were less common in patients with GC than in those without GC. The presence of contrast enhancement, necrosis, cystic change, hemorrhage, and GC type 2 were independent risk factors for predicting IDH mutation status in GC patients. GC remained as an independent prognostic factor (HR = 1.25, *P* = 0.031) in IDH-wildtype glioblastoma patients on multivariable analysis, along with clinical, molecular, and surgical factors. Overall, our data suggests that although no longer included as a distinct pathological entity in the WHO classification, recognition of GC may be crucial considering its clinical significance. There is a relatively high incidence of GC in adult-type diffuse gliomas, with different proportion according to molecular types between patients with and without GC. Imaging may preoperatively predict the molecular type in GC patients and may assist clinical decision-making. The prognostic role of GC promotes its recognition in clinical settings.

**Supplementary Information:**

The online version contains supplementary material available at 10.1186/s40478-024-01832-w.

## Introduction

Gliomatosis cerebri (GC) is defined as glioma showing a diffusely infiltrating growth pattern with the involvement of multiple contiguous lobes of the brain [27]. Although historically defined as an independent tumor entity in the 2007 World Health Organization (WHO) classification [27], subsequent studies showed that GC is a phenotypic growth pattern found in various types of diffuse gliomas rather than a separate entity [3, 8]. Thus, GC was excluded as a distinct tumor type in the 2016 WHO classification, as well as in the subsequent 2021 WHO classification [15, 16]. However, extensive infiltration of the brain with preservation of the local parenchymal architecture is a unique characteristic of GC that is different from the destructive and necrotic infiltrative pattern usually observed in high-grade gliomas [27, 28], and its clinical behavior is frequently neglected. However, due to its extremely invasive phenotype, GC is known to have poor prognosis within gliomas [7].

We have anecdotally noted in routine clinical practice that imaging patterns of GC seem to be relatively frequently encountered in adult-type diffuse gliomas. However, as previous studies have mostly focused on the subpopulation of patients with GC, its incidence within the entire population of adult-type diffuse glioma patients is unknown [8, 12, 25]. Moreover, the distribution of GC among the molecular types according to the recent 2021 WHO classification has not been reported; thus, whether a specific molecular type is prone to manifest as GC remains to be revealed. Due to the diffuse infiltrative growth pattern of GC, biopsy is often preferred over gross surgical removal; however, it frequently leads to nondiagnostic results due to cellular insufficiency [32]. Thus, the discovery of preoperative imaging phenotypes to predict the molecular type of GC may be crucial. Furthermore, whether GC is an important prognostic marker for more aggressive treatment planning also needs to be revealed.

We retrospectively collected data from pathologically confirmed adult-type diffuse glioma patients over a 17-year period at our institution and investigated the presence of GC. The aim of this study was to investigate the incidence, clinicopathologic and imaging correlates, and prognosis of GC in adult-type glioma patients according to the 2021 WHO classification.

## Materials and methods

**Fig. 1:**
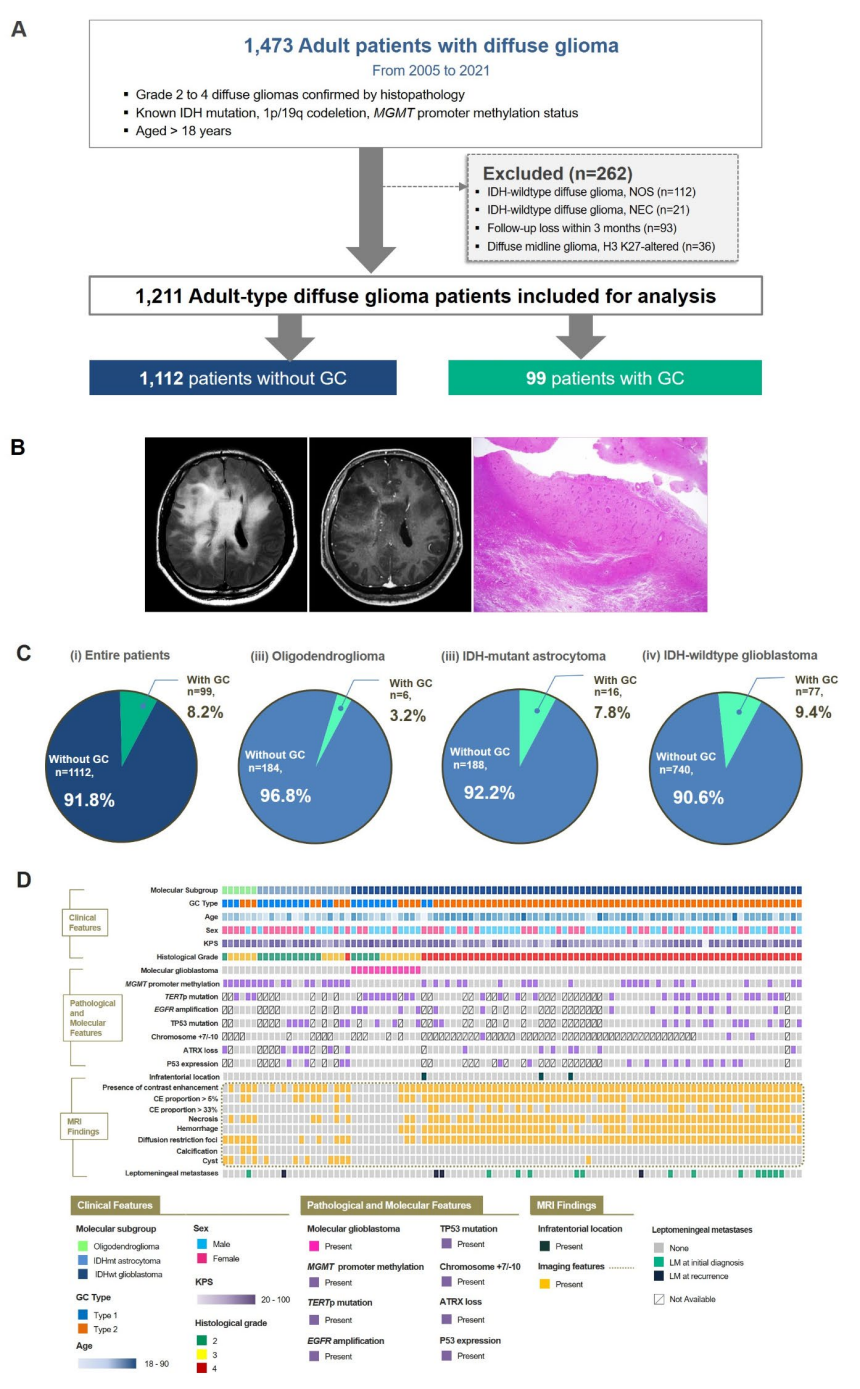
Patient characteristics of the study cohort of adult diffuse glioma patients of our institution. (**A**) Flow chart of patient inclusion. (**B**) Representative imaging and histologic findings in a patient with IDH-wildtype glioblastoma showing GC. On MRI, a diffuse infiltrative glioma involving bilateral cerebral hemispheres is seen on FLAIR image. Faint enhancement is seen in some areas on postcontrast T1-weighted image. On low-power view (H&E; x1.25), glioma cells are diffusely infiltrated into the cerebral parenchyma, suggesting GC. (**C**) Pie charts summarizing the distribution of molecular types of the adult-type diffuse glioma in patients with and without GC. (**D**) Summary plot of the clinical, molecular and imaging findings of patients with GC. GC = gliomatosis cerebri; IDH = isocitrate dehydrogenase; *MGMT* = O^6^-methylguanine-methyltransferase, NOS = not otherwise specified, NEC = not elsewhere classified, CE = contrast-enhancing, *TERT*p = telomerase reverse transcriptase promoter,  = epidermal growth factor receptor

The requirement for patient consent was waived owing to the retrospective study design from our institutional review board (Approval number: 4-2023-0045). A total of 1,473 consecutive patients with diffuse gliomas from our institution were included in this study. The inclusion criteria were as follows: (1) WHO grade 2 to 4 diffuse gliomas confirmed by histopathology, (2) known isocitrate dehydrogenase (IDH) mutation, 1p/19q codeletion, and O^6^-methylguanine-methyltransferase (*MGMT*) promoter methylation status, and (3) aged > 18 years. The exclusion criteria were as follows: (1) histological grade 2 or 3 IDH-wildtype diffuse gliomas that did not undergo testing for three genetic parameters (telomerase reverse transcriptase promoter [*TERT*p], epidermal growth factor receptor [*EGFR*] amplification, or combined gain of entire chromosome 7 and loss of entire chromosome 10 [+ 7/-10]), thereby diagnosed as IDH-wildtype diffuse glioma, not otherwise specified [17] (*n* = 112); (2) histological grade 2 or 3 IDH-wildtype diffuse gliomas that were negative for all three genetic parameters (*TERT*p, *EGFR*, and + 7/-10), thereby diagnosed as IDH-wildtype diffuse glioma, not elsewhere classified [17] (*n* = 21); (3) follow-up loss within 3 months (*n* = 93); and (4) presence of H3 K27M alteration, leading to a diagnosis of diffuse midline glioma, H3 K27-altered (*n* = 36). A total of 1,211 adult diffuse glioma patients were analyzed (Fig. 1A).

### Molecular classification

Patients were diagnosed according to the 2021 WHO classification [16]. All patients underwent IDH1/2 mutation, 1p/19q codeletion, and *MGMT* promoter methylation status testing. ATRX loss, p53 expression and *TERT*p mutation were also assessed. Since 2017, targeted next-generation sequencing (NGS) has been performed using the Illumina TruSight Tumor Panel. Apart from the traditional definition of IDH-wildtype glioblastoma, which is diagnosed based on the characteristic histological presence of microvascular proliferation or necrosis (“histological glioblastoma”), in the recent 2021 WHO classification, IDH-wildtype diffuse gliomas previously assigned to histological grade 2 or 3 can also be defined as IDH-wildtype glioblastoma in the presence of qualifying molecular markers (including *TERT*p mutation, *EGFR* gene amplification, and + 7/-10 (“molecular glioblastoma”). A total of 40 patients with histological grade 2 or 3 IDH-wildtype gliomas with either *TERT*p mutation, *EGFR* amplification, or + 7/-10 were classified as having IDH-wildtype glioblastoma, namely, ‘molecular glioblastoma’, according to the 2021 WHO classification [17]. ATRX loss, p53 protein expression, *TERT*p mutation, *EGFR* amplification, + 7/-10, and TP53 information was available for 1,026 (84.7%), 838 (69.2%), 868 (71.7%), 873 (72.1%), 469 (38.7%) and 870 (71.8%) patients, respectively. Details of the molecular classification can be found in Supplementary Material S1.

## MRI (magnetic resonance imaging) protocol

Brain MR images, including T1-weighted, T2-weighted, fluid-attenuated inversion recovery (FLAIR), postcontrast 3D T1-weighted, and diffusion-weighted images were acquired. The detailed parameters for the MRI protocols are listed in Supplementary Material S2.

### Image analysis

GC was diagnosed according to the following criteria: (1) T2-weighted or FLAIR imaging showing a diffuse process of infiltration involving at least three contiguous lobes and relative preservation of the anatomical architecture [8], and/or (2) pathological analysis confirming glial cell proliferation consistent with an infiltrative glioma. IDH-wildtype glioblastomas which have the dominant feature of a heterogeneous contrast-enhancing mass with mass effect and peritumoral edema were not diagnosed as GC if there was no underlying infiltrative non-enhancing tumor involving at least three contiguous lobes. Fig. 1B shows representative imaging and histological findings of GC.

All MRIs were reviewed and classified as either type 1 or type 2 GCs, according to the previous criteria [6]. Type 1 GCs are gliomas showing diffuse neoplastic growth and enlargement of the existing structure involved, without the formation of a discrete tumor mass at the initial clinical presentation. Type 2 GCs are gliomas with an obvious neoplastic mass, in addition to a diffuse infiltrative lesion involving more than three different lobes at the time of diagnosis (Supplementary Fig. 1) [19].

The location, presence of contrast enhancement, presence of necrosis, and presence of leptomeningeal metastases were identified [20]. Within GC patients, the presence of contrast enhancement, proportion of contrast-enhancing tumors > 5%, presence of diffusion restriction, cystic change, and hemorrhage were additionally labeled.

Bidimensional perpendicular measurement of the entire tumor was performed via preoperative and immediate postoperative imaging taken within 48-72 hour to evaluate the extent of resection (EOR) [10, 31]. The EOR was categorized as total (gross tumor removal, 100%), subtotal (≥ 75% but < 100%), partial (< 75%), or biopsy [10]. All imaging findings were reviewed by two neuroradiologists (Y.W.P. and S.S.A., with 11 and 18 years of experience, respectively) in consensus.

### Statistical analysis

#### Comparison of patient characteristics according to GC status

Patient characteristics were compared according to GC status in the entire adult-type diffuse glioma cohort using the chi-square test for categorical variables and the *t*-test or Mann‒Whitney *U* test for continuous variables, according to normality. Identical analysis were performed within subgroups of IDH-wildtype glioblastoma patients.

#### Prediction of IDH mutation status within GC

Logistic regression analyses were performed to predict IDH mutation status in GC patients. Variables of interest in the univariable analysis (*P* < 0.05) were included in the multivariable models using backward elimination according to the likelihood ratio with a variable selection criterion of *P* < 0.05. The area under the curve, accuracy, sensitivity, and specificity of the multivariable model were calculated.

#### Survival analysis of the entire adult-type diffuse glioma cohort and subgroup of IDH-wildtype glioblastoma patients

Survival rates were determined using the Kaplan‒Meier method, and curves were compared using the log-rank test for the entire cohort. The potential associations between the parameters and overall survival (OS) were evaluated by constructing univariable Cox proportional hazards regression models for each parameter. To assess whether GC remains an independent prognostic factor, multivariable Cox proportional hazards regression modeling for OS was performed. Identical analysis was performed within the subgroup of IDH-wildtype glioblastoma patients.

## Results

### Patient characteristics in the entire cohort according to GC status

This study included 1,211 adult diffuse glioma patients (age range: 18–90 years, median age: 56.4 years), comprising 509 females and 702 males, with a median follow-up period of 50.5 months (95% CI: 45.4–55.7). Among the 1,211 patients, 190 (15.7%) had oligodendroglioma, 204 (16.8%) had IDH-mutant astrocytoma, and 817 (67.5%) had IDH-wildtype glioblastoma. A total of 623 (51.4%) patients had *MGMT* promoter methylation.

There were 99 (8.2%) patients with GC among 1,211 adult-type diffuse glioma patients, six (3.2%) among the 190 oligodendroglioma patients, 16 (7.8%) among the 204 IDH-mutant astrocytoma patients, and 77 (9.4%) among the 817 IDH-wildtype glioblastoma patients. The proportion of molecular types was significantly different between patients with and without GC (*P* = 0.017) (Fig. 1C). A greater proportion of gliomas with GC than without GC were IDH-wildtype glioblastomas (77.8% vs. 66.5%), while oligodendrogliomas were observed in a smaller percentage of gliomas with GC than without GC (6.1% vs. 16.5%) (Fig. 1C). The proportion of patients with *MGMT* promoter methylation was lower (41.4% vs. 52.3%, *P* = 0.037) in patients with GC than in those without GC. Among the 1,026 (84.7%) patients in which data were available, the proportion of patients with ATRX loss was greater (26.1% vs. 16.3%, *P* = 0.019) among those with GC than among those without GC. According to the MRI findings, the proportion of patients with necrosis (70.7% vs. 48.5%, *P* < 0.001) was greater in patients with GC than in patients without GC. Gross total removal was not achieved in any patients with GC compared with those without GC (0% vs. 34.1%, *P* < 0.001). However, gross total resection of contrast-enhancing tumor was performed in 36 (36.4%) patients with GC, while biopsy was performed in 19 (19.2%) GC patients. The characteristics of the entire cohort and the patients stratified by the presence of GC are summarized in Table 1. Fig. 1D shows a summary plot of the clinical, molecular, and imaging findings of patients with GC.

**Table 1:** Characteristics in the adult-type diffuse glioma patients without and with GC

Characteristics	Total (*n* = 1,211)	Without GC (*n* = 1,112)	With GC (*n* = 99)	*P* ^*^
Age (year)	56.4 (29.2–76.6)	56.4 (29.2–76.8)	57.4 (28.7–73.5)	0.993
Sex (female)	509 (42.0)	464 (41.7)	45 (45.5)	0.471
KPS	90 (50–100)	90 (50–100)	80 (50–90)	**0.010**
CNS WHO grade				0.121
Grade 2	207 (17.1)	195 (17.5)	12 (12.1)	
Grade 3	160 (13.2)	151 (13.6)	9 (9.1)	
Grade 4	844 (69.7)	766 (68.9)	78 (78.8)	
Molecular classification				**0.017**
Oligodendroglioma	190 (15.7)	184 (16.5)	6 (6.1)	
IDH-mutant astrocytoma	204 (16.8)	188 (16.9)	16 (16.2)	
IDH-wildtype glioblastoma	817 (67.5)	740 (66.5)	77 (77.8)	
Other molecular markers				
IDH mutation	394 (32.5)	372 (33.5)	22 (22.2)	**0.022**
1p/19q codeletion	209 (17.3)	201 (18.1)	8 (8.1)	**0.011**
*MGMT* promoter methylation	623 (51.4)	582 (52.3)	41 (41.4)	**0.037**
*TERT*p mutation, present/tested	388/868 (44.7)	357/800 (44.6)	31/68 (45.6)	0.878
*EGFR* amplification, present/tested	187/873 (21.4)	173/802 (21.6)	14/71 (19.7)	0.715
+ 7/-10, present/tested^,^	72/469 (15.4)	69/430 (16.0)	3/39 (7.7)	0.166
TP53 mutation, present/tested^,^	210/870 (24.1)	190/802 (23.7)	20/68 (29.4)	0.290
ATRX loss, present/tested	176/1026 (17.2)	153/938 (16.3)	23/88 (26.1)	**0.019**
p53 protein expression	160/838 (19.1)	144/768 (18.8)	16/70 (22.9)	0.403
MRI findings				
Infratentorial location	37 (3.1)	34 (3.1)	3 (3.0)	0.973
Presence of contrast enhancement	949 (78.4)	865 (77.8)	83 (8.8)	0.141
Presence of necrosis	609 (50.3)	539 (48.5)	70 (70.7)	**< 0.001**
Leptomeningeal metastases	206 (17.0)	188 (16.9)	18 (18.2)	0.746
EOR of entire tumor				**< 0.001**
GTR	379 (31.3)	379 (34.1)	0 (0)	
Non-GTR	832(68.7)	733 (65.9)	99 (100)	
Death	638 (52.7)	573 (51.5)	65 (65.7)	**0.007**
Median OS (month)	33.1 (28.6–37.7)	35.3 (30.4–40.3)	16.7 (12.2–21.3)	**< 0.001**

Data are either median with 95% confidence interval or number with percentage in parentheses

* Calculated from Chi-square for categorical variables, and independent t-test or Mann-Whitney *U*-test for continuous variables according to normality

CNS = central nervous system; *EGFR* = epidermal growth factor receptor; EOR = extent of resection; GC = gliomatosis cerebri; GTR, gross total resection; IDH = isocitrate dehydrogenase; KPS = Karnofsky performance status; *MGMT* = O^6^-methylguanine-methyltransferase; OS = overall survival; *TERT*p = telomerase reverse transcriptase promoter; WHO = World Health Organization

### Patient characteristics in the subgroup of IDH-wildtype glioblastoma patients according to GC status

Among the 817 IDH-wildtype glioblastoma patients, 77 (9.4%) had GC. The proportion of histological grade 2 or 3 (molecular glioblastoma) patients was greater (15.6% vs. 3.8%, *P* < 0.001) in patients with GC than in those without GC. Among the 748 (91.6%) patients for whom data were available, the proportion of patients with ATRX loss was greater (18.9% vs. 9.9%, *P* = 0.018) among those with GC than among those without GC. Contrast enhancement was less frequent (89.6% vs. 96.2%, *P* = 0.001), while necrosis was more frequent (80.5% vs. 67.8%, *P* = 0.022) in patients with GC than in those without GC. The characteristics of the IDH-wildtype glioblastoma patients stratified by the presence of GC are summarized in Table 2.

**Table 2:** Characteristics in the IDH-wildtype glioblastoma patients without and with GC

Characteristics	Total (*n* = 817)	Without GC (*n* = 740)	With GC (*n* = 77)	*P* ^*^
Age (year)	61.7 (53.0–69.0)	62.0 (35.6–77.7)	60.0 (32.5–77.4)	0.070
Sex (female)	330 (40.4)	300 (40.5)	30 (39.0)	0.788
KPS	80 (70–90)	80 (50–100)	80 (40–90)	0.337
Histological grade 2 or 3 (Molecular glioblastoma)	40 (4.9)	28 (3.8)	12 (15.6)	**< 0.001**
Molecular markers				
*MGMT* promoter methylation	294 (36.0)	271 (36.6)	23 (29.9)	0.240
*TERT*p mutation, present/tested	308/613 (50.2)	280/558 (50.2)	28/55 (50.9)	0.918
*EGFR* amplification, present/tested	184/617 (29.8)	170/559 (30.4)	14/58 (24.1)	0.320
+ 7/-10, present/tested^,^	66/294 (22.4)	63/268 (23.5)	3/26 (11.5)	0.163
TP53 mutation, present/tested^,^	140/613 (22.8)	126/558 (22.6)	14/55 (25.5)	0.628
ATRX loss, present/tested	81/748 (10.8)	67/674 (9.9)	14/74 (18.9)	**0.018**
p53 protein expression	121/594 (20.4)	109/537 (20.3)	12/57 (21.1)	0.893
MRI findings				
Infratentorial location	32 (3.9)	29 (3.9)	3 (3.9)	0.992
Presence of contrast enhancement	781 (95.6)	712 (96.2)	69 (89.6)	**0.001**
Presence of necrosis	564 (69.0)	502 (67.8)	62 (80.5)	**0.022**
Leptomeningeal metastases	194 (23.7)	178 (24.1)	16 (20.8)	0.520
EOR of entire tumor				**< 0.001**
GTR	308 (37.7)	308 (41.6)	0 (0)	
Non-GTR	507 (62.1)	430 (58.1)	77 (100)	
Death	580 (71.0)	524 (70.8)	56 (72.7)	0.724
Median OS (month)	17.9 (16.7–19.1)	18.6 (17.3–20.0)	14.2 (11.9–16.4)	**0.002**

Data are either median with 95% confidence interval or number with percentage in parentheses

* Calculated from Chi-square for categorical variables, and independent t-test or Mann-Whitney *U*-test for continuous variables according to normality

*EGFR* = epidermal growth factor receptor; EOR = extent of resection; GC = gliomatosis cerebri; GTR = gross total resection; IDH = isocitrate dehydrogenase; KPS = Karnofsky performance status; *MGMT* = O^6^-methylguanine-methyltransferase; OS = overall survival; *TERT*p = telomerase reverse transcriptase promoter

### Risk factors for predicting IDH mutation status in patients with GC

Only 22 (22.2%) patients with GC had IDH mutation (6 with oligodendroglioma and 16 with IDH-mutant astrocytoma). The characteristics of the GC patients according to IDH mutation status are summarized in Supplementary Table 1. Compared with IDH-wildtype glioblastoma patients with GC, IDH-mutant patients with GC were significantly younger, had a greater proportion of females, less frequent contrast enhancement, a lower proportion of contrast-enhancing tumors (> 5%), less necrosis, less diffusion restriction, less hemorrhage, less frequent manifestation of GC type 2, and a greater proportion of cystic changes.

**Fig. 2:**
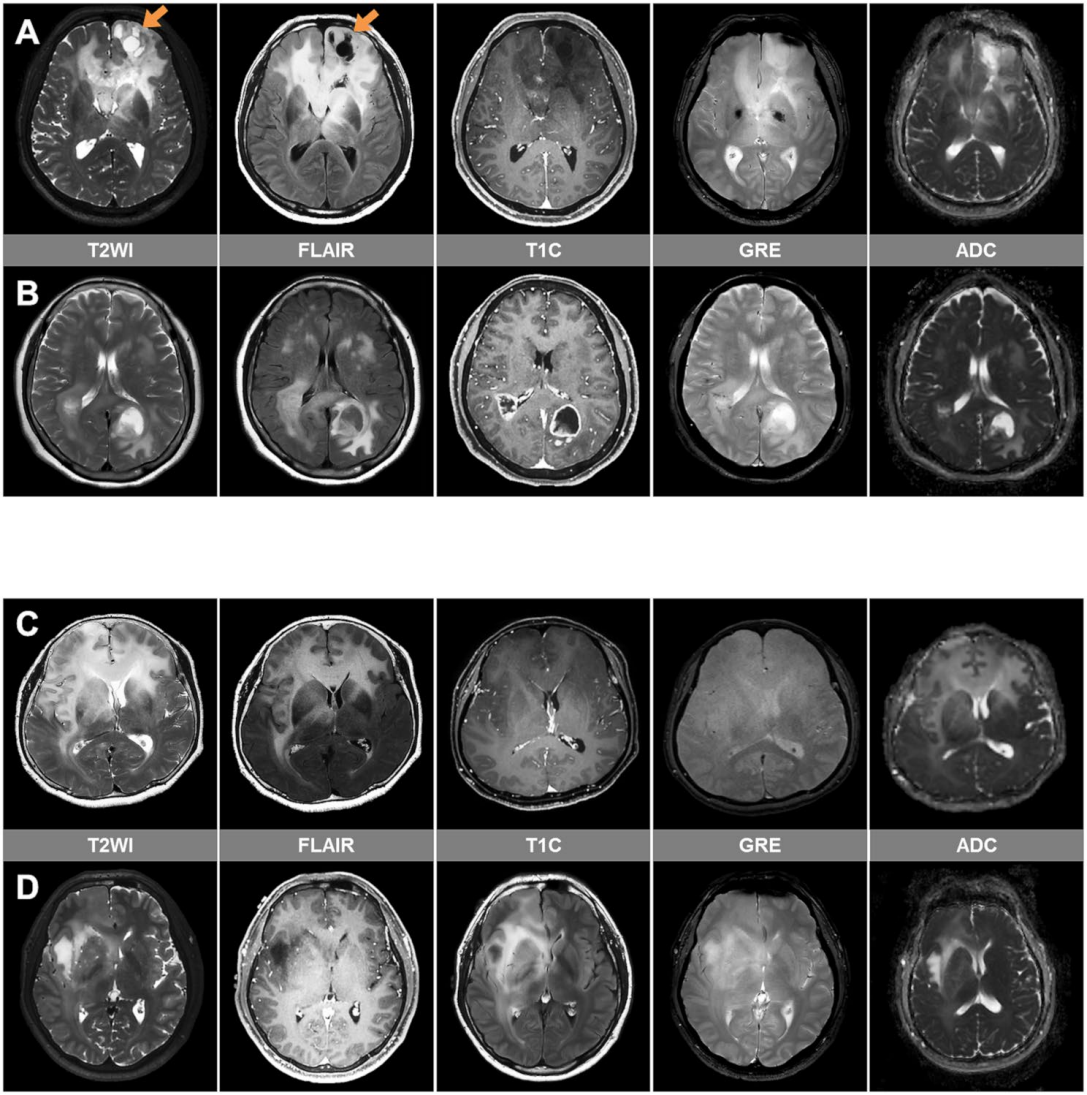
Representative imaging cases of GC cases with correctly (**A**, **B**) and incorrectly (**C**, **D**) predicted IDH mutation status according to multivariable model. (**A**) A 59-year-old male with IDH-mutant astrocytoma, CNS WHO grade 3. MRI shows a non-enhancing diffuse infiltrative tumor involving bilateral frontal lobes, left basal ganglia, and left thalamus. There is no discrete tumor mass, indicating type 1 GC. Cystic changes are seen at the left frontal lobe (arrows) on T2-weighted and FLAIR images. There is no hemorrhage on gradient recalled echo (GRE)-weighted image and no cellularity increase on apparent diffusion coefficient (ADC) map. (**B**) A 60-year-old female with IDH-wildtype glioblastoma, CNS WHO grade 4. MRI shows a non-enhancing diffuse infiltrative tumor involving the bilateral parietotemporooccipital lobes. There are obvious contrast-enhancing tumor masses, indicating type 2 GC. Contrast-enhancing necrotic tumor portions are seen at the right temporal and left parietotemporal lobes. There is a focal cellularity increase of solid enhancing tumor portions on ADC map. (**C**) A 65-year-old female with IDH-mutant astrocytoma, CNS WHO grade 2 showing a non-enhancing diffuse infiltrative tumor without necrosis, cystic change, nor hemorrhage. (**D**) A 32-year-old male with IDH-wildtype glioblastoma, CNS WHO grade 4. This patient was histologically grade 2, but was classified as IDH-wildtype glioblastoma due to presence of *TERT*p mutation (molecular glioblastoma). This case also shows imaging finding of a non-enhancing diffuse infiltrative tumor without necrosis, cystic change, nor hemorrhage

On multivariable analyses of clinical and imaging characteristics among GC patients, several imaging factors remained independent risk factors for predicting IDH mutation status, including the presence of contrast enhancement (odds ratio [OR] = 0.01, *P* < 0.001), necrosis (OR = 0.13, *P* < 0.001), cystic change (OR = 18.23, *P* < 0.001), hemorrhage (OR = 0.02, *P* < 0.001), and GC type 2 (OR = 0.08, *P* < 0.001) (Supplementary Table 2). The area under the curve, accuracy, sensitivity, and specificity of the multivariable model were 0.97 (95% CI 0.93-1.00), 87.9%, 86.4%, and 88.3%, respectively. Representative GC patients whose IDH mutation status were correctly predicted according to this model are shown in Fig. 2A and B. Among the 12 patients in which the multivariable model could not be used for accurate prediction, the MRI findings of all patients showed GC without contrast enhancement, necrosis, cystic, change, hemorrhage, or type 2 GC; 1 (8.3%) patient had grade 3 oligodendroglioma, and 3 (25%) patients had grade 2 IDH-mutant astrocytomas, while the remaining 8 (66.7%) patients had IDH-wildtype glioblastomas, which were histologically grade 2 or 3 (molecular glioblastoma). Representative cases of GC that were not correctly classified according to IDH mutation status are shown in Fig. 2C and D.

### Prognostic factors in the entire cohort according to GC status

**Fig. 3:**
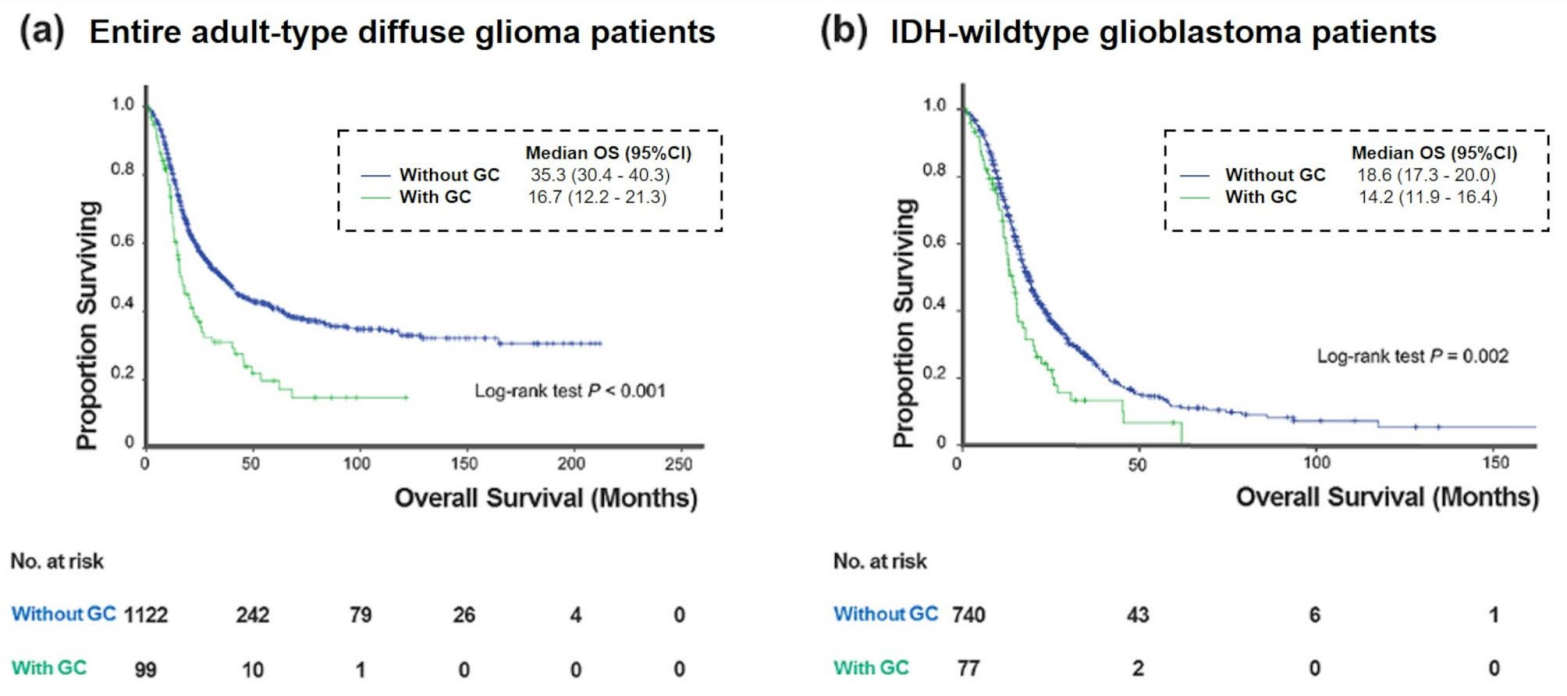
Kaplan-Meier curves of the OS of the according to the presence of GC in the (**a**) entire adult-type diffuse glioma patients and (**b**) IDH-wildtype glioblastoma patients. GC = gliomatosis cerebri; IDH = isocitrate dehydrogenase

On multivariable analysis, age, sex, Karnofsky Performance Scale (KPS), IDH mutation, 1p/19q codeletion, *MGMT* promoter methylation status, EOR, and leptomeningeal metastases were found to be independent predictors of OS. GC did not remain an independent predictor of OS (HR = 1.28, *P* = 0.083). The univariable and multivariable Cox analysis results are shown in Supplementary Table 3. The Kaplan–Meier curve showed that GC was a predictor of poor OS (log-rank test, *P* < 0.001; Fig. 3A). The median OS in patients with GC was 16.7 months (95% CI: 12.2–21.3), while the median OS in patients without GC was 35.3 months (95% CI: 30.4–40.3).

### Prognostic factors in subgroup of IDH-wildtype glioblastoma patients according to GC

According to multivariable GC analysis, GC status was an independent predictor of OS (HR = 1.25, *P* = 0.031), as were age, sex, KPS, *MGMT* promoter methylation status, presence of contrast enhancement, EOR, and leptomeningeal metastasis. The univariable and multivariable Cox analyses results are shown in Table 3. The Kaplan–Meier curve showed a significant difference in OS according to GC status (log-rank test, *P* = 0.002; Fig. 3B). The median OS in IDH-wildtype glioblastoma patients with GC was 14.2 months (95% CI: 11.9–16.4), while the median OS in IDH-wildtype glioblastoma patients without GC was 18.6 months (95% CI: 17.3–20.0).

**Table 3:** Univariable and multivariable cox analysis of risk factors for stratifying OS in IDH-wildtype glioblastoma patients with or without GC

Variables	Univariable		Multivariable	
	HR (95% CI)	*P* value	HR (95% CI)	*P* value
Older age^*^	1.02 (1.01–1.03)	**< 0.001**	1.03 (1.02–1.03)	**< 0.001**
Sex (female)	0.81 (0.68–0.95)	**0.012**	0.86 (0.79–0.94)	**0.002**
Higher KPS	0.98 (0.97–0.98)	**< 0.001**	0.98 (0.98–0.99)	**< 0.001**
*MGMT* promoter unmethylation	1.72 (1.44–2.05)	**< 0.001**	1.91 (1.60–2.29)	**< 0.001**
Infratentorial location	1.33 (0.90–1.95)	0.154		
Presence of contrast enhancement	2.05 (1.16–3.64)	**0.014**	1.91 (1.07–3.41)	**0.028**
Presence of necrosis	1.14 (0.96–1.37)	0.138		
Leptomeningeal metastases	1.34 (1.11–1.63)	**0.003**	1.34 (1.10–1.62)	**0.002**
GC	1.55 (1.18–2.05)	**0.002**	1.25 (0.94–1.03)	**0.031**
EOR		**< 0.001**		**< 0.001**
GTR	Reference	-	Reference	-
STR	1.77 (1.42–2.19)	**< 0.001**	1.91 (1.54–2.38)	**< 0.001**
PR	2.11 (1.71–2.60)	**< 0.001**	2.39 (1.93–2.96)	**< 0.001**
Biopsy	2.16 (1.62–2.88)	**< 0.001**	2.47 (1.85–3.29)	**< 0.001**

^*^An increase by 1 was considered when calculating ORs and 95% CIs

CI = confidence interval; EOR = extent of resection; GC = gliomatosis cerebri; GTR = gross total resection; HR = hazard ratio; IDH = isocitrate dehydrogenase; KPS = Karnofsky performance status; *MGMT* = O^6^-methylguanine-methyltransferase; OS = overall survival; PR = partial resection; STR = subtotal resection

## Discussion

This retrospective single-center study comprehensively examined the incidence, clinicopathologic and imaging correlates, and prognostic implications of GCs in adult-type diffuse gliomas. The estimated overall incidence of GC was 8.2% at our institution, which suggests that GC is relatively common in patients with adult-type diffuse gliomas. There was a greater proportion of IDH-wildtype glioblastomas and a lower proportion of oligodendrogliomas in gliomas with GC than in those without GC, suggesting that the manifestation of GC can help predict molecular markers. Moreover, imaging phenotypes reflect the underlying IDH mutation status in GC patients and thus can aid in the preoperative prediction of molecular markers. GC was a poor prognostic marker in the entire cohort suggesting that the preoperative identification of GC can aid in planning treatment for these patients. Overall, our results suggest that although GC is not a separate tumor entity, the relatively high incidence of GC in adult-type diffuse gliomas and its prognostic impact promote its recognition in clinical settings.

A recent study based on the SEER database reported that GC may represent 1/400 of glial tumors, and suggested its rare manifestation [7]. However, the results from population-based databases should be interpreted with caution since databases prevent direct access to imaging and recording of the data, which may include inconsistent labeling of GCs. Furthermore, this study did not exclusively examine the incidence of GC in patients with adult-type diffuse gliomas. Our data were labeled for the presence of GC by qualified neuroradiologists experienced in neuro-oncology with central review of imaging and showed that the incidence of GC was greater than expected, accounting for 8.2% of adult-type diffuse gliomas. Considering that one GC patient may appear among every twelve adult-type diffuse glioma patients, we propose that GC should not be neglected or missed but should be actively acknowledged on imaging.

Our results show that the proportion of GC is different among molecular types; IDH-wildtype glioblastomas manifest as GC more frequently, while oligodendrogliomas rarely manifest as GC. As IDH-wildtype glioblastomas are known to display high tumorigenicity and infiltrative migration [2], there may be a greater proportion of gliomas manifesting as GC. Notably, in our study, there was a significantly greater proportion of patients with IDH-wildtype gliomas with a histological grade of 2 or 3 (molecular glioblastoma) (15.6% vs. 3.8%, *P* < 0.001) among those with GC than among those without GC. Several previous studies have shown that IDH-wildtype glioblastoma patients with histological grade 2 or 3 (molecular glioblastoma) frequently have GC, which may support our findings [9, 13, 26]. The significantly lower proportion of *MGMT* promoter methylation in GC patients warrants further investigation, and the possibility of lower tumor purity in GC patients contributing to a false negative result in *MGMT* promoter methylation should be considered [14, 23]. However, as 36 (36.4%) GC patients underwent gross total resection of the contrast-enhancing tumor in case of presence of contrast-enhancing tumor portion, we speculate that the proportion of lower tumor purity should not be exceptionally high. A significantly higher frequency of ATRX loss was also observed in GC compared with patients without GC, both in IDH-mutant astrocytomas (*P* = 0.020, not shown) and IDH-wildtype glioblastomas (*P* = 0.018, Table 2), suggesting that ATRX loss may also play a unique role in the manifestation of GC. ATRX loss is reported to be associated with genomic instability and DNA damage [29], with increased cellular motility and invasion [5, 18]; however, the underlying molecular pathway of this association with GC should be explored in future studies.

Our results also showed that imaging phenotypes reflect the underlying IDH mutation status and can aid in the preoperative prediction of the IDH mutation status in patients with GC. Imaging features that predict the IDH mutation status in GC are in line with imaging features that generally predict IDH mutation status in glioma patients without GC; contrast enhancement, necrosis, absence of cystic change, and hemorrhage are all well-known imaging phenotypes for the differentiation of IDH-mutant gliomas from IDH-wildtype glioblastomas [22, 33]. Preoperative prediction of molecular features in GC patients may assist in planning treatment strategies and predicting the clinical course in patients with tissue insufficiency. The IDH mutation status in the majority of GC patients could be correctly classified according to imaging phenotypes, but some IDH-wildtype glioblastomas with histological grade 2 or 3 (molecular glioblastomas) tended to exhibit imaging features indistinguishable from those of IDH-mutant astrocytomas. It should be taken note of that in GC patients showing a non-enhancing diffuse infiltrative tumor without necrosis, cystic change, nor hemorrhage, there is a high probability of IDH-wildtype glioblastomas with a histological grade of 2 or 3 (molecular glioblastoma) (66.7%). As patients with molecular glioblastomas may consequently have identical biological behavior and prognosis as histological grade 4 IDH-wildtype glioblastomas this imaging manifestation should not be overlooked [1, 24]. Because the diagnosis of IDH-wildtype glioblastomas with histological grade 2 or 3 (molecular glioblastoma) is only possible after the post-NGS era, we speculate that the true proportion of IDH-wildtype glioblastomas with histological grade 2 or 3 within these non-enhancing GCs without any additional aggressive imaging phenotype may be even greater in future studies.

GC is known to have a dismal prognosis [4, 7, 25]; however, whether it is an independent prognostic factor along with other well-known clinical and molecular prognostic factors [11] in adult-type diffuse gliomas has not been evaluated. Our database had complete critical molecular information and meticulously labeled NGS results, imaging findings, EOR status, and the presence of leptomeningeal metastases in adult-type diffuse gliomas over the following period [11, 22, 34]. Although GC was not a significant prognostic factor in the multivariable analysis of the entire adult-type diffuse glioma cohort, it remained an independent prognostic factor in IDH-wildtype glioblastoma patients. Thus, special attention should be given to IDH-wildtype glioblastoma with GC, especially when the optimal treatment strategy is yet to be revealed [35].

There are several limitations in our study. First, our study analyzed a single center, retrospectively collected dataset. Second, data on *EGFR* amplification, *TERT*p mutation, and + 7/-10 status were available only at a particular period [21]. Thus, only a small portion of patients with histological grade 2 or 3 IDH-wildtype gliomas with molecular features of glioblastomas were enrolled. Third, all GC lesions cannot be evaluated via histopathological examination because gross total removal cannot be achieved in GC patients, possibly resulting in intratumoral heterogeneity. However, in routine practice, neurosurgeons target the area with most aggressive imaging findings on surgery, which may decrease the possibility of histological downgrading of GC patients. Lastly, post-surgical treatments were not included in the analysis. However, all patients received standard adjuvant therapy in accordance with the recommended EANO guidelines, tailored to each patient’s molecular type of tumor, WHO grade, age, and performance [30]. 

## Conclusion

The incidence of GC is 8.2% in patients with adult-type diffuse gliomas, with a greater proportion of patients with IDH-wildtype gliomas than patients without GC. Preoperative imaging features may predict IDH mutation status in GC patients. GC is an independent marker of poor prognosis in IDH-wildtype glioblastomas. Thus, it becomes important to reorganize and discuss GCs in imaging reports as well as in multidisciplinary tumor boards.

## Electronic supplementary material

Below is the link to the electronic supplementary material.

Supplementary Material 1

## Data Availability

Data generated or analyzed during the study are available from the corresponding author by request.

## Abbreviations

GC: Gliomatosis cerebri
WHO: World Health Organization
IDH: Isocitrate dehydrogenase
*MGMT*: O^6^-methylguanine-methyltransferase
*TERT*p: Telomerase reverse transcriptase promoter
*EGFR*: Epidermal growth factor receptor
NGS: Next-generation sequencing
MRI: Magnetic resonance imaging
FLAIR: Fluid-attenuated inversion recovery
EOR: Extent of resection
OS: Overall survival
KPS: Karnofsky performance status
