# Correction: Revisiting gliomatosis cerebri in adult-type diffuse gliomas: a comprehensive imaging, genomic and clinical analysis

**DOI:** 10.1186/s40478-024-01869-x

**Published:** 2024-11-06

**Authors:** Ilah Shin, Yae Won Park, Yongsik Sim, Seo Hee Choi, Sung Soo Ahn, Jong Hee Chang, Se Hoon Kim, Seung-Koo Lee, Rajan Jain

**Affiliations:** 1 Department of Radiology, Seoul St. Mary’s Hospital, College of Medicine, The Catholic University of Korea, 222 Banpo-daero, Seocho-gu, Seoul, 06591 Republic of Korea; 2 Department of Radiology and Research Institute of Radiological Science and Center for Clinical Imaging Data Science, College of Medicine, Yonsei University, 50 Yonsei-ro, Sedaemun-gu, Seoul, 03722 Republic of Korea; 3 Department of Radiation Oncology, Yonsei University College of Medicine, 50 Yonsei-ro, Sedaemun-gu, Seoul, 03722 Republic of Korea; 4 Department of Neurosurgery, Yonsei University College of Medicine, 50 Yonsei-ro, Sedaemun-gu, Seoul, 03722 Republic of Korea; 5 Department of Pathology, Yonsei University College of Medicine, 50 Yonsei-ro, Sedaemun-gu, Seoul, 03722 Republic of Korea; 6 Department of Radiology, New York University Grossman School of Medicine, 550 1 Ave, New York, NY States USA; 7 Department of Neurosurgery, New York University Grossman School of Medicine, 550 1 Ave, New York, NY States USA

**Correction: Acta Neuropathologica Communications (2024) 12:128** 10.1186/s40478-024-01832-w

Following publication of the original article [1], the author found that the affiliation details for author Ilah Shin were incorrectly given as Ilah Shin^1,2^ but should have been Ilah Shin^1^. The second affiliation, “Department of Statistics and Data Science, Yonsei University, 50 Yonsei-ro, Sedaemun-gu, Seoul, 03722, Republic of Korea” has been deleted, and the affiliation order has been renumbered. And, there is a misalignment in Fig. 1 and the resolution of figures 2 and 3 needs to be improved.

Incorrect Figure 1
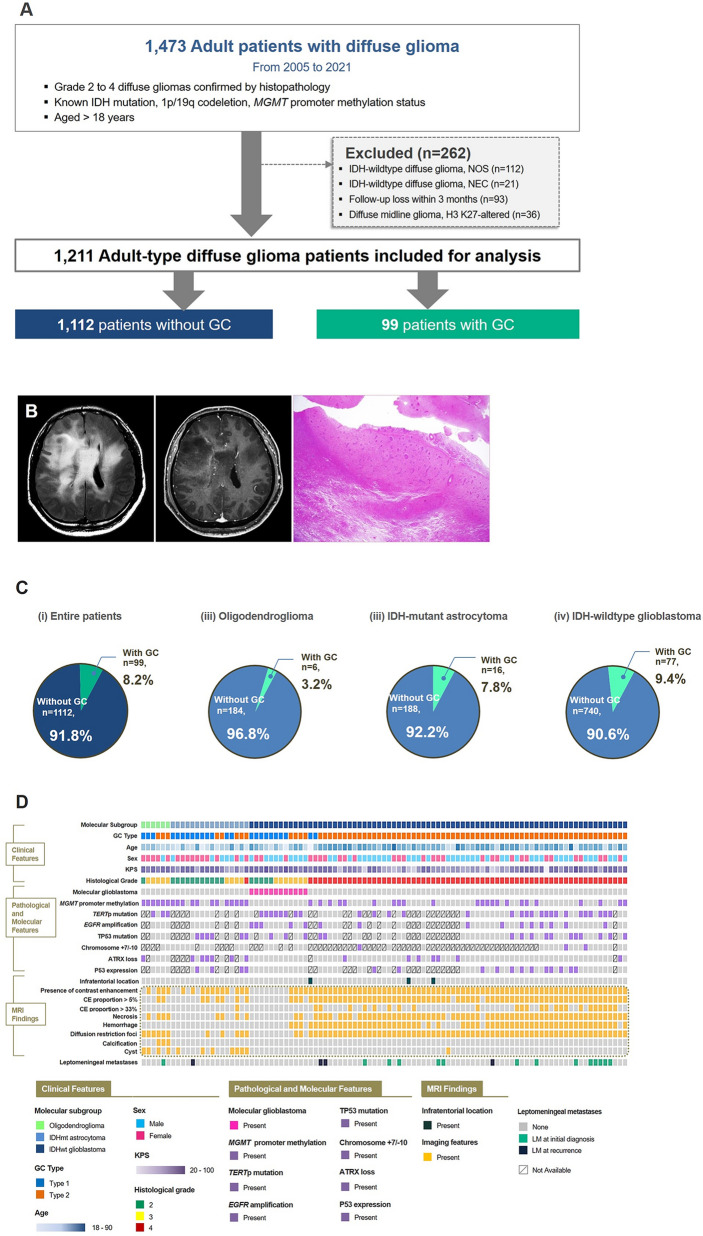


The figures should have appeared as shown below.

**Fig. 1:**
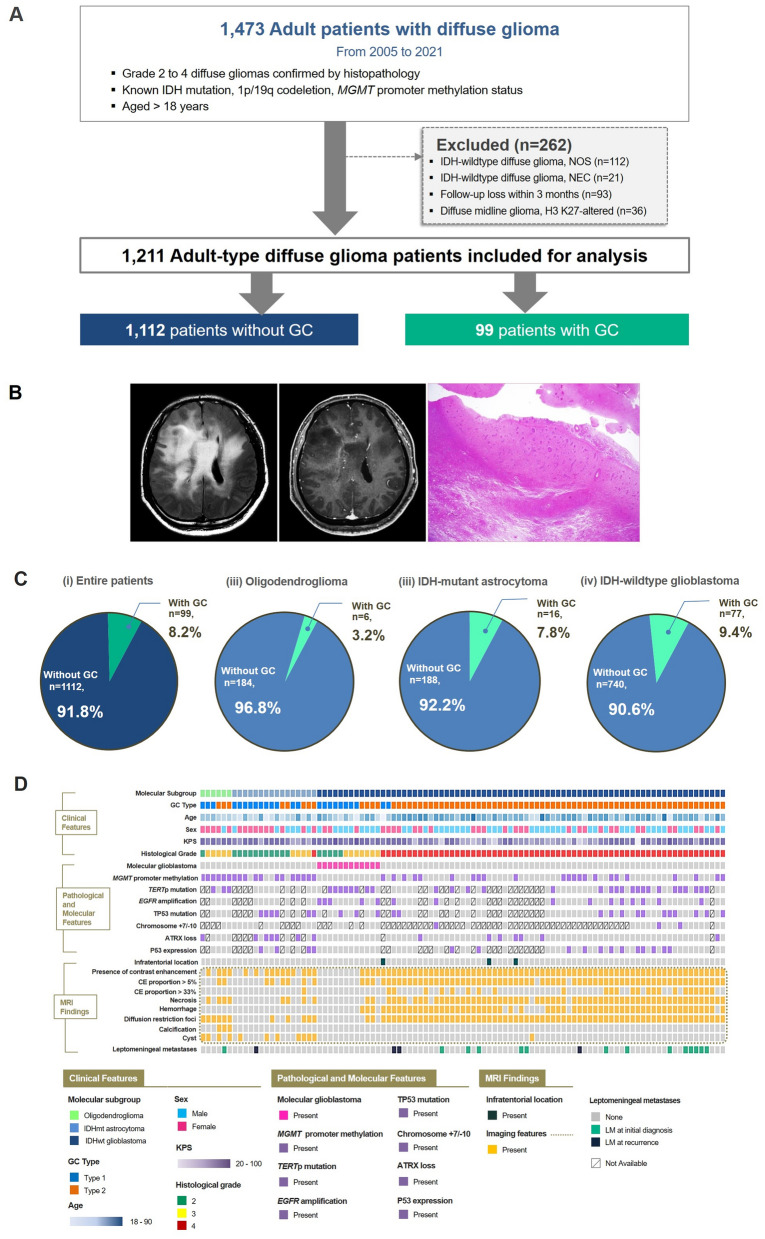
Patient characteristics of the study cohort of adult diffuse glioma patients of our institution. **A** Flow chart of patient inclusion. **B** Representative imaging and histologic findings in a patient with IDH-wildtype glioblastoma showing GC. On MRI, a diffuse infiltrative glioma involving bilateral cerebral hemispheres is seen on FLAIR image. Faint enhancement is seen in some areas on postcontrast T1-weighted image. On low-power view (H&E; × 1.25), glioma cells are diffusely infiltrated into the cerebral parenchyma, suggesting GC. **C** Pie charts summarizing the distribution of molecular types of the adult-type diffuse glioma in patients with and without GC. **D** Summary plot of the clinical, molecular and imaging findings of patients with GC. GC = gliomatosis cerebri; IDH = isocitrate dehydrogenase; *MGMT* = O6-methylguanine-methyltransferase, NOS = not otherwise specified, NEC = not elsewhere classified, CE = contrast-enhancing, *TERT*p = telomerase reverse transcriptase promoter, = epidermal growth factor receptor

**Fig. 2:**
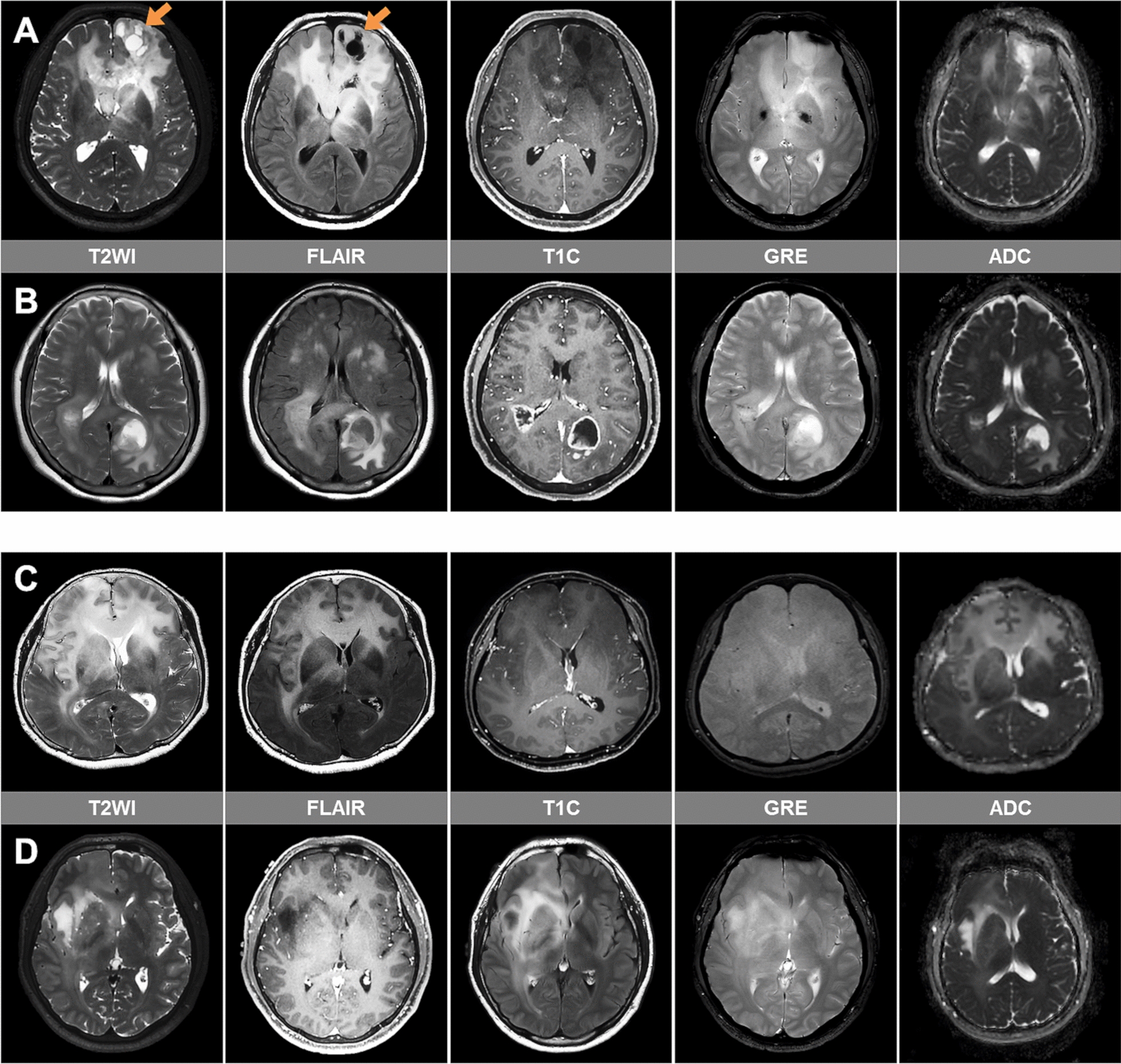
Representative imaging cases of GC cases with correctly **A**, **B** and incorrectly **C**, **D** predicted IDH mutation status according to multivariable model. **A** A 59-year-old male with IDH-mutant astrocytoma, CNS WHO grade 3. MRI shows a non-enhancing diffuse infiltrative tumor involving bilateral frontal lobes, left basal ganglia, and left thalamus. There is no discrete tumor mass, indicating type 1 GC. Cystic changes are seen at the left frontal lobe (arrows) on T2-weighted and FLAIR images. There is no hemorrhage on gradient recalled echo (GRE)-weighted image and no cellularity increase on apparent diffusion coefficient (ADC) map. **B** A 60-year-old female with IDH-wildtype glioblastoma, CNS WHO grade 4. MRI shows a non-enhancing dif-fuse infiltrative tumor involving the bilateral parietotemporooccipital lobes. There are obvious contrast-enhancing tumor masses, indicating type 2 GC. Contrast-enhancing necrotic tumor portions are seen at the right temporal and left parietotemporal lobes. There is a focal cellularity increase of solid enhancing tumor portions on ADC map. **C** A 65-year-old female with IDH-mutant astrocytoma, CNS WHO grade 2 showing a non-enhancing diffuse in-filtrative tumor without necrosis, cystic change, nor hemorrhage. **D** A 32-year-old male with IDH-wildtype glioblastoma, CNS WHO grade 4. This patient was histologically grade 2, but was classified as IDH-wildtype glioblastoma due to presence of *TERT*p mutation (molecular glioblastoma). This case also shows imaging finding of a non-enhancing diffuse infiltrative tumor without necrosis, cystic change, nor haemorrhage

**Fig. 3:**
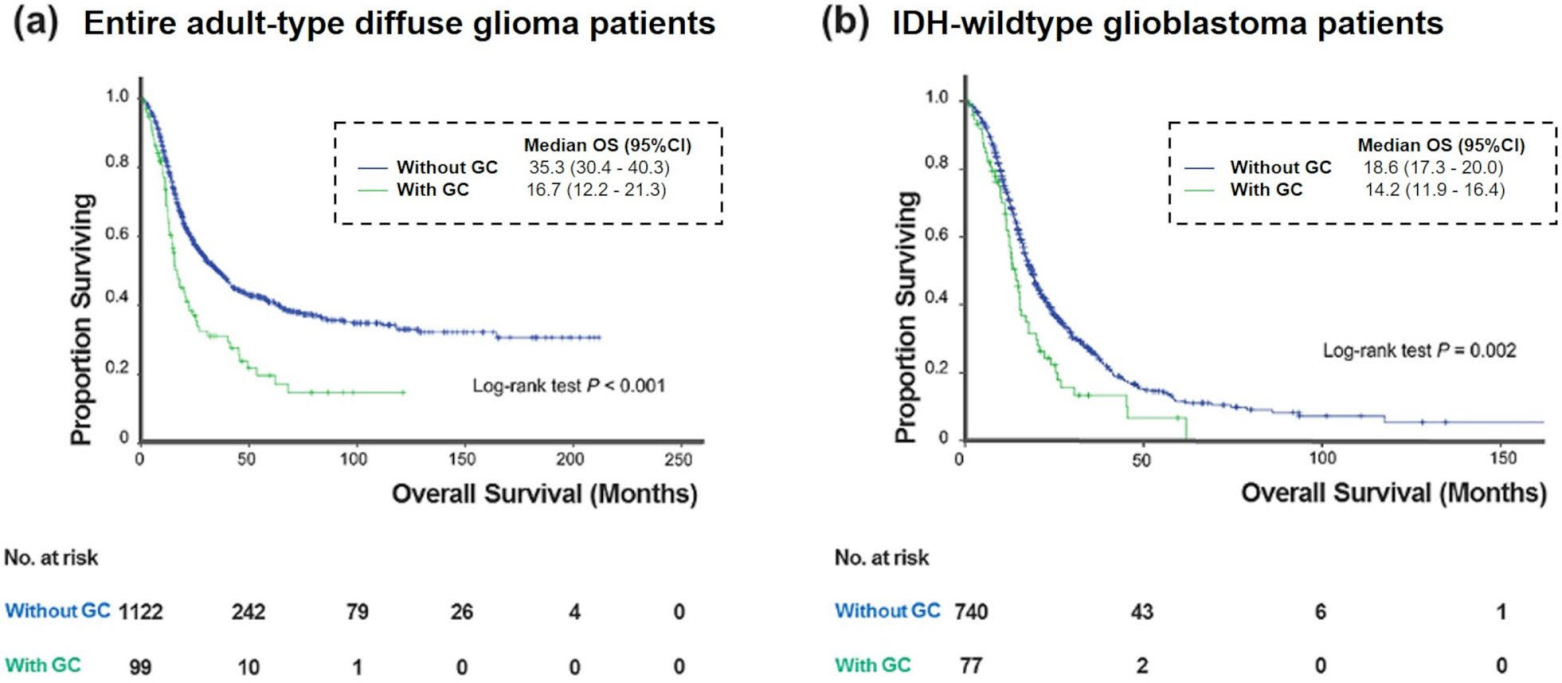
Kaplan–Meier curves of the OS of the according to the presence of GC in the **a** entire adult-type diffuse glioma patients and **b** IDH-wildtype glioblastoma patients. GC = gliomatosis cerebri; IDH = isocitrate dehydrogenase

The original article has been corrected.
