# Supplementary material for: Revisiting gliomatosis cerebri in adult-type diffuse gliomas: a comprehensive imaging, genomic and clinical analysis
Source: Acta Neuropathol Commun. 2024 Aug 10;12:128. doi: 10.1186/s40478-024-01832-w (PMC11316408; doi:10.1186/s40478-024-01832-w)
Supplement: Supplementary file 1 — Supplementary Material 1 [file 40478_2024_1832_MOESM1_ESM.docx]

**Supplementary Materials.**

**S1. Molecular Classification**

All tumors were diagnosed according to the 2021 WHO classification.^1^ Both immunohistochemical (IHC) analysis and peptide nucleic acid-mediated clamping polymerase chain reaction were performed to detect IDH1 R132H mutation. In IDH1-negative patients on IHC analysis, IDH1/2 status was confirmed by peptide nucleic acid-mediated clamping polymerase chain reaction. Fluorescent in situ hybridization analysis was performed to detect 1p/19q codeletion. The presence of histone H3 K27M mutant protein was evaluated by IHC analysis using polyclonal antibodies to detect the histone H3.3 tail. MGMT promoter methylation was evaluated by methylation-specific polymerase chain reaction.^2^ All patients underwent IDH1/2 and MGMT promoter methylation status testing.

ATRX alterations were assessed by IHC analysis. Cases with more than 10% positive tumor cells were scored positive, whereas cases with less than 10% positive tumor cells were scored as ATRX loss. p53 protein expression was assessed by IHC analysis, and > 50% of the nuclei stained was considered as positive of p53 expression.^3^ For copy number analysis, TERTp mutation was determined using a pyrosequencing assay, and C228T and C250T mutations were analyzed.

Since 2017, targeted next-generation sequencing (NGS) was performed using the Illumina TruSight Tumor 170 panel.^4^ For copy number analysis, EGFR genes with ≥ 2 fold-change relative to the average level were considered to have undergone amplification. Total 40 patients with histological grade 2 or grade 3 IDH-wildtype gliomas with either TERTp mutation, EGFR gene amplification, or chromosome +7/-10 were classified as IDH-wildtype glioblastoma, according to the 2021 WHO classification.^5^

**S2. MRI protocol**

Brain magnetic resonance imaging (MRI) including T1-weighted, T2-weighted, pre- and postcontrast fluid-attenuated inversion recovery (FLAIR), postcontrast 3D T1-weighted images, and diffusion weighted images were acquired in a 3T unit. Spine MRI including axial and sagittal postcontrast T1-weighted images, were acquired with a 1.5T.

A 3T MRI unit (Achieva or Ingenia; Philips Healthcare) and an 8-channel sensitivity encoding head coil were used for brain MRI. The protocol included T1-weighted turbo spin-echo images with inversion recovery (repetition time [TR], 2000 ms; echo time [TE], 10 ms; inversion time [TI], 1000 ms; field of view [FOV], 240 x 240 mm; section thickness, 5 mm; matrix, 256 × 256), T2-weighted turbo spin-echo (TR, 3000 ms; TE, 80 ms; FOV, 240 x 240 mm; section thickness, 5 mm; matrix, 256 × 256), and T2-weighted fluid-attenuated inversion recovery (FLAIR) (TR, 10,000 ms; TE, 125 ms; TI, 2500 ms; FOV, 240 x 240 mm; section thickness, 5 mm; matrix, 256 × 256) images. Postcontrast 3D T1-weighted turbo field echo images (TR, 9.8 ms; TE, 4.6 ms; FOV, 240 x 240 mm; section thickness, 1 mm; matrix, 224 × 224) was acquired 6 minutes after the injection of gadolinium-based contrast (0.1 mL/kg of gadobutrol, Gadovist; Bayer Schering Pharma, Berlin, Germany). Postcontrast FLAIR (TR, 10,000-11,000 ms; TE, 110-125 ms; TI, 2500 ms; square FOV, 230-240 mm; section thickness, 5 mm; matrix, 256 × 256) was subsequently acquired 10 minutes and 30 seconds after the injection of gadolinium-based contrast. Diffusion weighted image was acquired with the following parameters: b values of 1000 s/mm2 and 0 s/mm2, 3 directions, TR, 8000-12,000; TE, 60-80 ms; FOV, 240 x 240 mm, section thickness, 5 mm; matrix, 256 × 256.

Spine MRI was obtained using a 1.5T unit (Achieva dStream; Philips Healthcare). The protocol included sagittal T1-weighted (TR, 450 ms; TE, 9.8 ms; section thickness, 3 mm; matrix: 314 × 448; FOV, 350 mm), axial T1-weighted (TR, 520 ms; TE, 9.3 ms; section thickness, 3 mm; matrix, 202 x 384; FOV, 250 mm), axial T2-weighted (TR, 3430 ms; TE, 120 ms; section thickness, 3 mm; matrix, 202 x 384; FOV, 250 mm), and sagittal T2-weighted (TR, 3760 ms; TE, 100 ms; section thickness, 3 mm; matrix, 338 × 512; FOV, 350 mm) images. Post-contrast T1-weighted images in axial and sagittal planes were acquired after the injection of gadolinium-based contrast (0.1 mL/kg of gadobutrol, Gadovist; Bayer Schering Pharma, Berlin, Germany).

Supplementary Figure 1. Representative imaging of Type 1 and Type 2 GCs. (a) Type 1 GC showing infiltrative tumor growth involving more than three different lobes and without discrete mass formation. (b) Type 2 GC shows a contrast-enhancing mass with background infiltrative tumor.


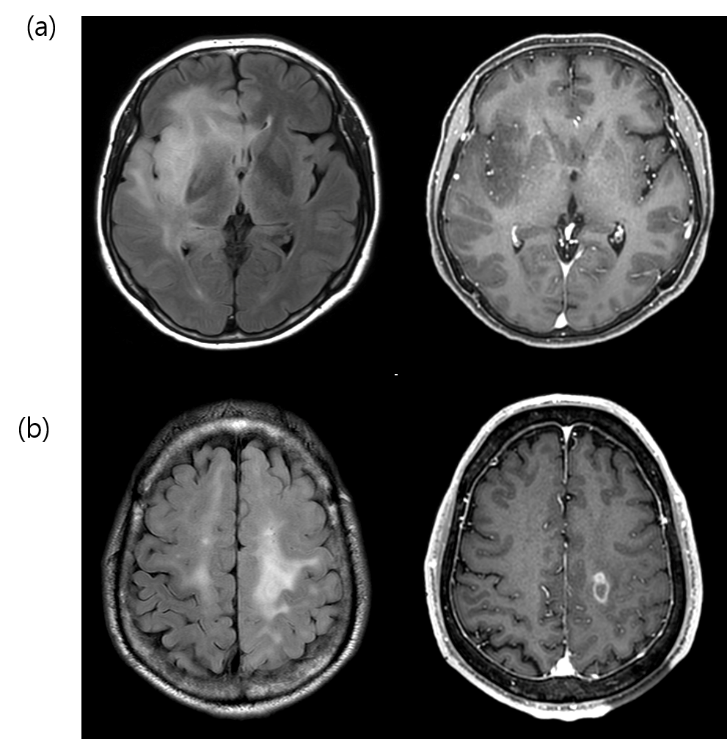


Supplementary Table 1. Clinical and imaging characteristic in GC patients according to IDH mutation status.

| Characteristics | IDH-mutant (n = 22) | IDH-wildtype (n = 77) | *P*^*^ |
| --- | --- | --- | --- |
| Age (year, 95% CI) | 42.4 (24.6-67.0) | 60.0 (32.5-77.4) | **< 0.001** |
| Sex (female) | 15 (68.2) | 30 (39.0) | **0.015** |
| GC type 2 | 8 (36.4) | 67 (87.0) | **< 0.001** |
| Infratentorial location | 0 (0) | 3 (3.9) | 0.347 |
| Presence of  contrast enhancement | 15 (68.2) | 69 (89.6) | **0.013** |
| Proportion of CE tumor > 5% | 8 (36.4) | 66 (85.7) | **< 0.001** |
| Necrosis | 8 (36.4) | 62 (80.5) | **< 0.001** |
| Diffusion restriction | 11 (50.0) | 68 (88.3) | **< 0.001** |
| Cystic change | 11 (50.0) | 1 (1.3) | **< 0.001** |
| Calcification | 3(13.6) | 0 (0) | **0.001** |
| Hemorrhage | 0 (0) | 60 (77.9) | **< 0.001** |

Data are either median with 95% confidence interval or number with percentage in parentheses.

* Calculated from Chi-square for categorical variables, and independent t-test or Man-Whitney U-test for continuous variables according to normality.

CE = contrast-enhancing; CI =confidence interval; GC = gliomatosis cerebri; IDH = isocitrate dehydrogenase

Supplementary Table 2. Univariable and multivariable logistic analysis of risk factors forpredicting IDH mutation status in GC patients.

| Variables | Univariable | | Multivariable | |
| --- | --- | --- | --- | --- |
|  | OR (95% CI) | *P* | OR (95% CI) | *P* |
| Age (year) | 0.93 (0.89-0.97) | **< 0.001** | - | - |
| Sex (female) | 3.36 (1.23-9.19) | **0.018** | - | - |
| GC type 2 | 0.09 (0.03-0.26) | **< 0.001** | 0.08 (0.04-0.22) | **< 0.001** |
| Infratentorial location | na | - |  |  |
| Presence of contrast enhancement | 0.25 (0.08-0.79) | 0.018 | 0.01 (0.00-0.04) | **< 0.001** |
| Proportion of CE tumor > 5% | 0.10 (0.03-0.28) | **< 0.001** | - | - |
| Necrosis | 0.14 (0.05-0.39) | **< 0.001** | 0.13 (0.04-0.37) | **< 0.001** |
| Diffusion restriction | 0.15 (0.05-0.43) | **< 0.001** | - | - |
| Cystic change | 76.00 (8.92-647.60) | **< 0.001** | 18.23 (2.52-75.82) | **< 0.001** |
| Hemorrhage | 0.02 (0.01-0.04) | 0.997 | 0.02 (0.01-0.03) | **< 0.001** |

CE = contrast enhancing; CI =confidence interval; GC = gliomatosis cerebri; OR = odds ratio

Supplementary Table 3. Univariable and multivariable cox analysis of risk factors for stratifying OS in adult-type glioma patients with or without GC.

| Variables | Univariable | | Multivariable | |
| --- | --- | --- | --- | --- |
|  | HR (95% CI) | *P* value | HR (95% CI) | *P* value |
| Older age * | 1.05 (1.04-1.06) | **< 0.001** | 1.03 (1.02-1.03) | **< 0.001** |
| Sex (female) | 0.78 (0.67-0.92) | **0.003** | 0.87 (0.80-0.94) | **0.002** |
| Higher KPS | 0.97 (0.96-0.97) | **< 0.001** | 0.98 (0.98-0.99) | **< 0.001** |
| CNS WHO grade 4 | 12.81(9.52-17.24) | **< 0.001** | - | - |
| IDH-wildtype | 13.24 (9.98-17.58) | **< 0.001** | 3.73 (2.56-5.40) | **< 0.001** |
| No 1p/19q codeletion | 6.72 (4.74-9.52) | **< 0.001** | 1.75 (1.18-2.61) | **0.005** |
| MGMT promoter unmethylation | 3.35 (2.83-3.95) | **< 0.001** | 1.93 (1.63-2.30) | **< 0.001** |
| Infratentorial location | 1.78 (1.23-2.63) | **0.002** | - | - |
| Presence of contrast enhancement | 8.26 (5.91-11.55) | **< 0.001** | 1.97 (1.34-2.88) |  |
| Presence of necrosis | 3.07 (2.60-3.63) | **< 0.001** | - | - |
| GC | 1.81 (1.40-2.35) | **< 0.001** | 1.28 (0.97-1.70) | 0.083 |
| EOR of entire tumor |  | **< 0.001** |  | **< 0.001** |
| GTR | Reference | - |  |  |
| STR | 1.87 (1.52-2.29) | **< 0.001** | 2.04 (1.65-2.51) | **< 0.001** |
| PR | 2.19 (1.80-2.66) | **< 0.001** | 2.47 (2.02-3.03) | 0.284 |
| Biopsy | 3.29 (2.48-4.36) | **< 0.001** | 2.54 (1.90-3.40) | 0.941 |

^*^An increase by 1 was considered when calculating ORs and 95% CIs

CI = confidence interval; EOR = extent of resection; GC = gliomatosis cerebri; GTR = gross total resection; HR = hazard ratio; IDH = isocitrate dehydrogenase; MGMT = O^6^-methylguanine-DNA methyltransferase; OS = overall survival; PR = partial resection; STR = subtotal resection

**References**

1. Louis DN, Perry A, Wesseling P, et al. The 2021 WHO Classification of Tumors of the Central Nervous System: a summary. Neuro Oncol 2021;23:1231-1251.

2. Esteller M, Garcia-Foncillas J, Andion E, et al. Inactivation of the DNA-repair gene MGMT and the clinical response of gliomas to alkylating agents. N Engl J Med 2000;343:1350-1354.

3. Birner P, Piribauer M, Fischer I, et al. Prognostic relevance of p53 protein expression in glioblastoma. Oncol Rep 2002;9:703-707.

4. Park YW, Park JE, Ahn SS, et al. Magnetic Resonance Imaging Parameters for Noninvasive Prediction of Epidermal Growth Factor Receptor Amplification in Isocitrate Dehydrogenase-Wild-Type Lower-Grade Gliomas: A Multicenter Study. Neurosurgery 2021;89:257-265.

5. Louis DN, Wesseling P, Paulus W, et al. cIMPACT-NOW update 1: Not Otherwise Specified (NOS) and Not Elsewhere Classified (NEC). Acta Neuropathol 2018;135:481-484.
